# Association of Various Optical Coherence Tomographic Patterns of Diabetic Macular Edema With Central Subfield Thickness and Visual Acuity: A Cross-Sectional Observational Study

**DOI:** 10.7759/cureus.69731

**Published:** 2024-09-19

**Authors:** Abinaya Ramakrishnan, Nithya R., Divya N., Panimalar A Veeramani

**Affiliations:** 1 Ophthalmology, Saveetha Medical College and Hospitals, Saveetha Institute of Medical and Technical Sciences, Saveetha University, Chennai, IND

**Keywords:** central subfield thickness, diabetic macular edema (dme), diabetic retinopathy, optical coherence tomography, patterns, visual acuity

## Abstract

Introduction

Diabetic macular edema (DME) is the most common and vision-threatening complication in diabetic patients with diabetic retinopathy (DR), especially in those with Type 2 diabetes mellitus. Optical coherence tomography (OCT) is a reliable tool most commonly used for assessing macular morphology and provides quantitative information on the macula. OCT also examines the outer retinal layers, which can predict visual outcomes. Thus, our study aims to identify the association of various OCT-detected DME morphological patterns with central subfield thickness (CST) and visual acuity.

Materials and methods

This is a cross-sectional observational study of 50 patients with DME detected on OCT who visited the Ophthalmology Department of Saveetha Medical College and Hospitals for a period of six months, from November 2023 to April 2024. A complete ocular examination, including best corrected visual acuity, scored with the logMAR scale, anterior segment examination, and fundus biomicroscopy using 90D and 78D lenses, was performed. Early Treatment of Diabetic Retinopathy Study (ETDRS) grading of DR into mild to very severe non-proliferative diabetic retinopathy (NPDR) and proliferative diabetic retinopathy (PDR) was noted. Spectral-domain OCT was used to diagnose DME. The CST was measured, and DME was classified into four patterns: sponge-like retinal swelling (SLRS), cystoid macular edema (CME), subretinal fluid (SRF), and posterior hyaloid traction (PHT).

Results

In the present study, males represented 60%, and females represented 40%. The mean age of the patients was 58.07 ± 6.80 years, with a mean duration of diabetes of 11.91 ± 5.14 years. Of the 50 patients with 100 eyes, only 60 eyes showed DME on OCT. CME was the most common morphological pattern (37%), while the least common pattern was PHT (10%). No significant association was found between a specific morphological pattern and control of diabetes. The most common pattern observed was SLRS in moderate NPDR, CME in severe NPDR, SRF in very severe NPDR, and PHT in PDR. Very severe NPDR patients showed all patterns of DME, and the PHT pattern was observed only in very severe NPDR and PDR. The highest mean CST was observed in the very severe NPDR stage, and the least was in the moderate NPDR stage. The mean CST was highest in SRF patterns and lowest in SLRS patterns. The best mean visual acuity was observed in the SLRS pattern, while the worst mean visual acuity was observed in the SRF pattern, followed by the PHT pattern.

Conclusion

Our study highlights the importance of OCT in patients with diabetes, as OCT patterns of DME are critical for predicting visual outcomes in DR. Severe grades of DR are usually associated with SRF and PHT patterns. Since patients with SRF and PHT patterns have the worst visual outcomes, these patients, upon identification, need to be counseled about their poor visual prognosis. Those with less severe DR should be closely monitored and advised on effective diabetes control to prevent progression and protect their vision.

## Introduction

Diabetic macular edema (DME) is the most prevalent and significant vision-related consequence of diabetic retinopathy (DR) in developed nations, particularly among individuals with Type 2 diabetes mellitus (DM) [1]. Estimates suggest that the global prevalence of DME in both Type 1 DM and Type 2 DM is 7.48%, and it can affect up to 15% of individuals two decades after diagnosis [1-3]. With DR usually affecting the working-age population, it adds to the burden on the community. DME leads to visual loss, as it causes swelling of the area responsible for high-resolution visual acuity in the macula. Multiple factors, including metabolic, inflammatory, and vascular elements, contribute to the pathogenesis of DME [4].

The vascular elements include vascular endothelial growth factor (VEGF), which plays a crucial role in the occurrence of DME and is overexpressed in diabetic patients. Higher levels of VEGF have been noted in both the vitreous and retina in patients with DR [5]. These factors deteriorate the blood-retinal barrier by acting on the tight junctions of retinal endothelial cells, primarily due to chronic elevation of blood sugar levels, abnormal blood lipid levels, increased damage from reactive oxygen species, and persistent low-level inflammation. As a result, fluid and protein leak into the macula, causing DME [6].

Optical coherence tomography (OCT) is a reliable tool for high-resolution, cross-sectional images of retinal layers. It is also the most frequently used method in clinical practice to assess macular morphology, abnormalities in the vitreomacular interface, and quantitative information such as macular thickness, macular volume, the integrity of the external limiting membrane (ELM), and the inner segment-outer segment (IS-OS) junction, all of which significantly influence final visual acuity in DME [7]. OCT also examines the outer retinal layers, which predict the visual outcome [8]. Thus, the role of OCT has become significant in the assessment and management of DR and the associated DME.

It has been reported that central retinal thickening is inversely related to visual acuity in DME patients. If the value of macular thickness is normalised in these patients, they may recover partial visual function [9]. Since different morphological subtypes of macular edema may play a significant role in the differences in visual acuity observed in DME patients, our study aims to identify the association of various OCT-detected DME morphological patterns with central subfield thickness (CST) and visual acuity.

## Materials and methods

Study design

Ethical clearance was obtained from the Institutional Ethics Committee of Saveetha Medical College and Hospitals. This cross-sectional study was conducted on 50 diabetic patients with DME who visited the Outpatient Department of Ophthalmology for a period of six months, from November 2023 to April 2024, after obtaining informed written consent.

Inclusion criteria

Diabetic patients with NPDR or PDR, with clinically evident DME on OCT, were included in the study.

Exclusion criteria

Patients with active proliferative retinopathy, with vitreous hemorrhage or dense media haze that made it difficult to obtain OCT images, patients with eye diseases other than DR that can cause vision loss and macular edema, recent ocular surgery, and patients with other macular pathologies, glaucoma, and high refractive errors were excluded.

Data collection

A detailed patient's history of diabetes duration, treatment, and other comorbidities was obtained. Laboratory investigations, including hemoglobin A1c (HbA1c), fasting blood sugar (FBS), and postprandial blood sugar (PPBS), were done to evaluate the control of diabetes. A complete ophthalmic examination, including the logMAR score for best corrected visual acuity, anterior segment slit lamp examination, and fundus biomicroscopy with 90D and 78D lenses, was performed. After the fundus examination, the Early Treatment of Diabetic Retinopathy Study (ETDRS) grading of DR into mild to very severe non-proliferative diabetic retinopathy (NPDR) and proliferative diabetic retinopathy (PDR) was noted. Spectral-domain OCT was used to diagnose DME. No eyes with mild NPDR were noted to have DME and, hence, were not included in our study. Fovea-centered macular scans with a quality greater than 4/10 for each eye were selected, and the CST was measured. Based on OCT imaging and classifications used in previous studies, including Ahmadpour-Baghdadabad et al., we classified these images into four distinct patterns: sponge-like retinal swelling (SLRS), cystoid macular edema (CME), subretinal fluid (SRF), and posterior hyaloid traction (PHT). These four patterns, using cross-sectional OCT pictures of retinal structure reflectivity, were analysed [10].

In this study, instances with both SLRS and CME were deemed CME. Any SLRS or CME pattern associated with SRF was included in the SRF pattern. Vitreomacular tractions (VMTs) or vitreomacular interface abnormalities (VMIAs) were considered PHT, regardless of the other pattern combinations involved.

Statistical analysis

The software Microsoft Excel (Microsoft® Corp., Redmond, WA, USA) was used to store and structure the data for statistical analysis with IBM SPSS Statistics for Windows, Version 25 (Released 2017; IBM Corp., Armonk, NY, USA). Mann-Whitney U test, Chi-square test, and analysis of variance (ANOVA) were the various methods used in the present study. A p-value of <0.05 was considered statistically significant.

## Results

Clinical characteristics of the study subjects

In the present study, males were 30 in number (60%), and females were 20 in number (40%). Among the 50 patients included in the study, four patients (8%) belonged to the 41-50 years age group, 32 patients (64%) were in the age group of 51-60 years, 11 patients (22%) were in the age group of 61-70 years, and three patients (6%) were over 70 years old. The mean age of patients was 58.07 ± 6.80 years. Using the Mann-Whitney test, age in male and female patients was analysed and found no significance (p-value: 0.374).

The duration of DM in the studied population was less than five years in six patients (12%), 6-10 years in 11 patients (22%), 11-15 years in 23 patients (46%), 16-20 years in seven patients (14%), and more than 20 years in three patients (6%), with a mean duration of diabetes of 11.91 ± 5.14 years.

Of the 50 patients with 100 eyes, only 60 eyes showed the presence of DME on OCT. Four different patterns of DME were found upon evaluation of the OCT scans. Specifically, 14 patients (23%) had SLRS patterns, 22 patients (37%) had CME patterns, 18 patients (30%) had SRF patterns, and six patients (10%) had PHT patterns. CME was the most common morphological pattern (37%), while the least common pattern was PHT (10%).

Association of DME patterns with control of diabetes

Among the 60 eyes, the association of various patterns of DME and the control of diabetes was noted. No significant association was found between a specific morphological pattern and the control of diabetes, as can be seen in Table 1.

**Table 1:** Association of various patterns with control of diabetes using Chi-square test *p > 0.05 is statistically insignificant SLRS: Sponge-like retinal swelling; CME: Cystoid macular edema; SRF: Subretinal fluid; PHT: Posterior hyaloid traction

Diabetic macular edema patterns	Control of diabetes		p-value
	Uncontrolled N(%)	Controlled N(%)	
SLRS	5 (25%)	9 (75%)	0.124*
CME	10 (45.5%)	12 (54.5%)	
SRF	12 (66.7%)	6 (33.3 %)	
PHT	5 (87.5%)	1 (12.5%)	

Association of DME patterns with grades of DR

Among the 60 eyes, the association of different grades of DR and various patterns of DME was analysed. The most common pattern observed was SLRS in moderate NPDR, CME in severe NPDR, SRF in very severe NPDR, and PHT in PDR. Very severe NPDR patients showed all patterns of DME, and the PHT pattern was observed only in very severe NPDR and PDR, as can be seen in Table 2.

**Table 2:** Association of grades of diabetic retinopathy with various patterns of diabetic macular edema using analysis of variance test *p < 0.05 is statistically significant SLRS: Sponge-like retinal swelling; CME: Cystoid macular edema; SRF: Subretinal fluid; PHT: Posterior hyaloid traction; NPDR: Non-proliferative diabetic retinopathy; PDR: Proliferative diabetic retinopathy

Grade of diabetic retinopathy	SLRS	CME	SRF	PHT	Total	p-value
Moderate NPDR	8 (66.6%)	3 (25%)	1 (8.3%)	0	12	<0.001*
Severe NPDR	3 (12.5%)	14 (58.3%)	7 (29.1%)	0	24	
Very severe NPDR	2 (11.7%)	4 (23.5%)	10 (58.8%)	1 (5.8%)	17	
PDR	1 (14.2%)	1 (14.2%)	0	5 (71.4%)	7	
Total	14	22	18	6	60	

Association of grades of DR with mean CST

The mean CST among various stages of DR was 380 microns in moderate NPDR, 484.96 microns in severe NPDR, 552.47 microns in very severe NPDR, and 398.14 microns in the PDR group. The highest mean CST was observed in the very severe NPDR stage, while the least was in the moderate NPDR stage, as can be seen in Table 3.

**Table 3:** Association of mean central subfield thickness with various grades of diabetic retinopathy using analysis of variance test *p < 0.05 is statistically significant PDR: Proliferative diabetic retinopathy; NPDR: Non-proliferative diabetic retinopathy

Grade of diabetic retinopathy	Number of eyes N(%)	Mean central subfield thickness (microns)	p-value
Moderate NPDR	12 (20%)	380	<0.001*
Severe NPDR	24 (40%)	484.96	
Very severe NPDR	17 (28.3%)	552.47	
PDR	7 (11.7%)	398.14	

Association of DME patterns with mean CST

The mean CST in various morphological patterns was 372.07 microns in SLRS, 424.32 microns in the CME pattern, 536.78 microns in the SRF pattern, and 446.83 microns in the PHT pattern. The mean CST was highest in the SRF patterns and lowest in the SLRS patterns, as can be seen in Table 4.

**Table 4:** Association of mean central subfield thickness with various patterns of DME using analysis of variance test *p < 0.05 is statistically significant SLRS: Sponge-like retinal swelling; CME: Cystoid macular edema; SRF: Subretinal fluid; PHT: Posterior hyaloid traction; DME: Diabetic macular edema

Diabetic macular edema patterns	Number of eyes	Mean central subfield thickness (microns)	p-value
SLRS	14	372.07	<0.001*
CME	22	424.32	
SRF	18	536.78	
PHT	6	446.83	

Association of DME patterns with mean visual acuity

The mean visual acuity among the 60 eyes was observed to be 0.54 logMAR units. Mean visual acuity of 0.426, 0.512, 0.742, and 0.680 logMAR units was observed among SLRS, CME, SRF, and PHT patterns, respectively. The best mean visual acuity was observed in the SLRS pattern, while the worst mean visual acuity was observed in the SRF pattern (0.742 logMAR), as can be seen in Table 5.

**Table 5:** Association of mean visual acuity with various patterns of DME using analysis of variance test *p < 0.05 is statistically significant SLRS: Sponge-like retinal swelling; CME: Cystoid macular edema; SRF: Subretinal fluid; PHT: Posterior hyaloid traction; DME: Diabetic macular edema

Diabetic macular edema patterns	Number of eyes	Mean visual acuity (logMAR units)	p-value
SLRS	14	0.426	<0.001*
CME	22	0.512	
SRF	18	0.742	
PHT	6	0.68	

## Discussion

In our study, the number of males (30) exceeded the number of females (20), which is in accordance with many studies where male preponderance was noted [11,12]. The mean age of our patients was 58.07 ± 6.80 years, which is comparable to studies by Hannouche et al. and Starr et al., where the mean age was 59.3 ± 10.2 years and 55.6 ± 8.86 years, respectively [3,13]. The mean duration of diabetes among the patients was 11.91 ± 5.14 years, which is similar to the findings of Ahmadpour-Baghdadabad et al., where the mean age was 12.21 ± 6.1 years [10].

The most common morphological pattern observed in our study was the CME pattern, accounting for 37% of cases. However, a study done by Qureshi et al. found that the SLRS pattern is the most prevalent pattern observed [14]. PHT is the least frequently observed pattern in our study, accounting for just 14% of individuals, as noted in a study by Lee et al. [15].

It is known that longstanding diabetes and poor glycemic control are significant risk factors for DME development [16]. However, no significant association between a specific morphological DME pattern and the control of diabetes was noted in our study. In our study, only a few cases of PDR were observed, which is similar to other studies in the literature [17,18]. This could be due to regular screening of diabetic patients and increased healthcare facilities.

Patients can develop DME at any stage of DR, but its prevalence increases with the severity of DR [19]. In our study, DME prevalence was higher in severe NPDR (40%) than in very severe NPDR (28.3%) and PDR (11.7%). This could also be attributed to routine screening, which might lead patients to present at an earlier stage. However, the patterns of DME associated with different stages of DR varied significantly. Moderate and severe NPDR usually showed SLRS and CME patterns, while very severe NPDR showed more of the SRF pattern, and the majority of PDR patients showed the PHT pattern. The PHT pattern was noted to be observed only in very severe NPDR and PDR.

Our study showed that the SRF patterns had the highest mean CST, while the SLRS patterns had the lowest mean CST. These findings were in concordance with Pokharel et al.'s study, where the highest average CST was observed in the SRF pattern and the lowest average CST in the SLRS pattern [20]. However, in their study, the PHT pattern was not documented, probably because the sample consisted only of eyes that showed NPDR with clinically significant macular edema (CSME).

Better visual acuity in our study was noted in the SLRS pattern, while the worst visual outcome was observed in the SRF and PHT patterns. Similar to our study, Hannouche et al. also noted better visual acuity with SLRS patterns, while Qureshi et al. noted the worst visual acuity with PHT patterns [3,14].

With increasing CST, the visual acuity worsens, as there is a significant linear relation between CST and visual acuity [21,22]. Our study also demonstrated that the patterns with higher CST values had poorer visual acuity. OCT, with this quantitative measurement of CST in characterising DME, could help with early DME identification and more targeted treatments [23].

However, since the present study is a cross-sectional, hospital-based study, the results cannot be generalised. Additionally, the present study used only the CST measured by OCT to identify the association of DME patterns with visual acuity. With the advent of newer OCT biomarkers, including disruption of the ellipsoid zone (EZ) and the presence of hyperreflective foci, further studies are required to identify a reliable OCT biomarker for association with visual acuity.

## Conclusions

DME is a vision-threatening complication of DR, and our study highlights the role of OCT in identifying the different patterns of DME and the association of various OCT patterns with visual outcomes in patients. The different grades of DR and the various patterns of DME that were associated with increased CST showed poor visual acuity, confirming the linear relationship between CST and visual acuity. The best visual prognosis was noted in patients with SLRS patterns. Severe grades of DR are usually associated with SRF and PHT patterns, which, in turn, have the worst visual outcomes. Hence, all diabetic patients must be regularly screened, and patients with SRF and PHT patterns need to be counseled about their poor visual prognosis. Additionally, patients with less severe DR should be followed up regularly and advised on adequate control of diabetes to prevent progression to worse visual outcomes.
